# A VigiBase Descriptive Study of Fluoroquinolone-Associated Peripheral Nervous System Disorders

**DOI:** 10.3390/ph15020143

**Published:** 2022-01-26

**Authors:** Madalina Huruba, Andreea Farcas, Daniel Corneliu Leucuta, Camelia Bucsa, Cristina Mogosan

**Affiliations:** 1 Department of Pharmacology, Physiology and Physiopathology, Faculty of Pharmacy, “Iuliu Haţieganu” University of Medicine and Pharmacy, 400349 Cluj-Napoca, Romania; huruba.madalina@umfcluj.ro (M.H.); cmogosan@umfcluj.ro (C.M.); 2 Drug Information Research Center, “Iuliu Hatieganu” University of Medicine and Pharmacy, 400349 Cluj-Napoca, Romania; cfarah@umfcluj.ro; 3 Department of Medical Informatics and Biostatistics, “Iuliu Hatieganu” University of Medicine and Pharmacy, 400349 Cluj-Napoca, Romania; dleucuta@umfcluj.ro

**Keywords:** fluoroquinolones, VigiBase, peripheral neuropathy, safety concerns

## Abstract

Background: Recent drug safety concerns described fluoroquinolone (FQ)-induced peripheral nervous system reactions. The objective of this study was to characterize such reports from VigiBase. Methods: The analysis included FQ-induced peripheral nervous system disorder adverse drug reaction (ADR) reports (up to July 2019). We looked into the disproportionality data in terms of proportional reporting ratio (PRR) and information component (IC) values, and descriptive analysis was performed for FQ-ADRs positive associations (ADRs, suspected FQs, potential risk factors, such as associated therapy and underlying disease). Results: Disproportionality analysis revealed 4374 reports (3531 serious) with peripheral nervous system ADRs associated with at least three FQs (neuropathy peripheral, 5492; neuralgia, 481; polyneuropathy, 220; sensory loss, 99; peripheral sensorimotor neuropathy, 39). Among these, both time-to-onset and duration of reaction were mostly between 1–7 days and ≥30 days. Most of the ADRs were not recovered/resolved at the time of reporting. Conclusion: The results augment the existing data on FQ safety concerns, specifically their potential effect on the nervous system.

## 1. Introduction

Widely used for both prophylaxis and the therapy of various infections [1,2], due to their high antibacterial activity [1,3], broad spectra (against respiratory, genitourinary, gastrointestinal, bone, and ophthalmic infections), and favorable pharmacokinetics [3], fluoroquinolones (FQs) are generally well tolerated [2,4,5,6], reportedly possessing a favorable safety profile [7]. Nevertheless, serious, disabling, and potentially permanent side effects associated with the class and affecting muscles, joints, and the nervous system, triggered safety reviews by both the U.S. Food and Drug Administration (FDA) (2016) [8] and the European Medicines Agency (EMA) (2019) [9]. These resulted in restrictions of use and downgrade of (fluoro)quinolones from being the first-line antibacterial agents, with specific exceptions [10].

A matter of public concern [6], the safety reviews focused on FQ-associated disability (FQAD) involving the nervous system, as well as tendons, muscles, and joints [10], the first of which represents the topic of the present research, particularly the peripheral nervous system. FQ-associated musculoskeletal disability has been previously described [11]. Historically, after gastrointestinal symptoms, nervous system disturbances are among the most reported adverse drug reactions (ADRs) from FQs [2,4,5,12,13,14]. A systematic review found that the occurrence of central nervous system (CNS)-related ADRs (along gastrointestinal [GI]) was significantly higher with FQs compared to other antimicrobials [15]. Such reactions vary in severity and include headache, dizziness, agitation, sleep disorders, psychoses, and, rarely, convulsions [13].

In the United States (US), in 2004, peripheral neuropathy (PN) was added to product information and medication guidelines as an identified risk of systemic treatment along with drug class [16,17,18,19,20]. An optimal characterization of the risk was further requested by the FDA in 2013 [21]. Important aspects such as seriousness, onset, or reversibility of PN, as well as the development of acute nerve damage (including Guillain-Barré syndrome [GBS]) among individual FQ agents, remain uncertain [1]. This is similar to other nervous system disorders that may arise as ADRs, depending on the individual FQ [2,4,5,12,13].

Quinolone-associated neurotoxicity was first mentioned in the literature 40 years ago [22]. To date, ADRs affecting the peripheral and CNS are included in the quinolone product information, and thus, well recognized; however, limited data quantifying both relative and absolute risk of PN from FQ exposure is available [23].

The objective of this retrospective study was to characterize individual case safety reports (ICSRs) with an FQ as the suspected drug, resulting in peripheral nervous system disorders, from VigiBase, the unique World Health Organization (WHO) global database of ICSRs.

## 2. Results

**Figure 1:**
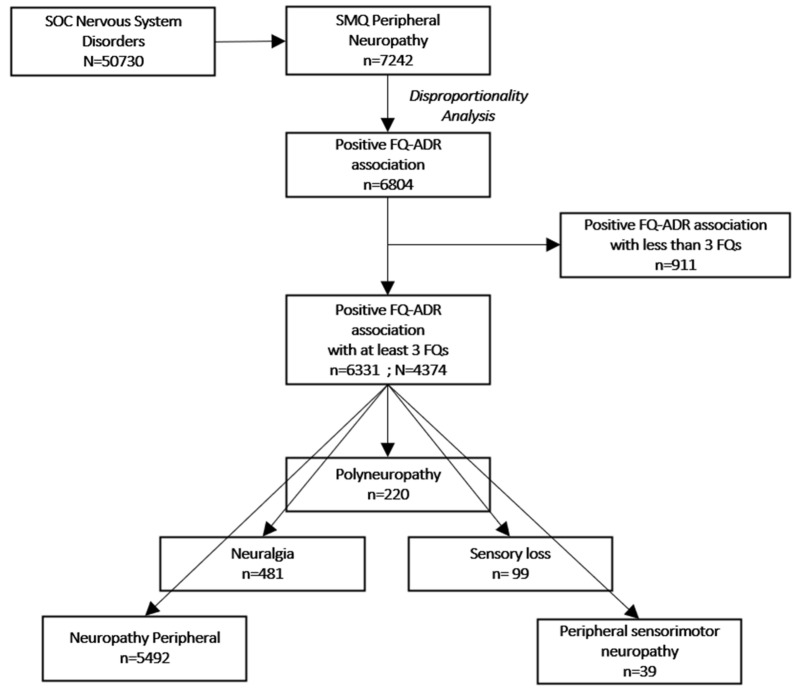
Flowchart of selection criteria. ADR: adverse drug reaction; FQ: fluoroquinolone; SOC: system organ class (MedDRA); SMQ: standardized MedDRA queries; N: number of reports; n: number of adverse drug reactions.

Our descriptive analysis included 4374 unique reports containing 6331 FQ-ADR positive associations with at least three FQs, as resulted from the disproportionality analysis (Figure 1).

### 2.1. Disproportionality Analysis

We looked at ADRs (in terms of PTs) pertaining to PN Standardized MedDRA Query (SMQ) [24], among which, only neuropathy peripheral, neuralgia, peripheral sensorimotor neuropathy, polyneuropathy, and sensory loss were the ones associated with at least three FQs. Descriptive analysis was performed on the 4374 individual reports, which contained 6331 peripheral nervous system disorder ADRs positively associated with at least three FQs (Table 1).

A similar approach was used for GBS SMQ [24], as we intended to include this data too in our analysis. A total of 72 associations were depicted for FQs and GBS, however, PRR and IC values did not fulfil the criteria for a positive association: ciprofloxacin: IC025 = −0.81, PRR025 = 0.59; levofloxacin: IC025 = −1.35, PRR025 = 0.41; moxifloxacin: IC025 = −2.04, PRR025 = 0.27; norfloxacin: IC025 = −2.84, PRR025 = 0.17; ofloxacin: IC025 = −2.42, PRR025 = 0.21. Therefore, no further analysis was employed on this particular dataset.

### 2.2. General Characteristics

**Figure 2:**
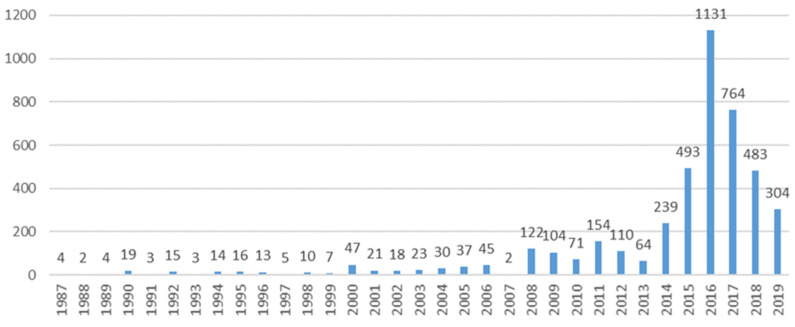
Year trends of reporting.

Among the total 4374 individual reports associated with 6331 ADRs, the female sex prevailed among all age groups, with adults being the topmost affected patients (Table 2). A peak in reporting rates was noted in 2016 (Figure 2).

From the total 4374 unique reports, 3531 were serious, 556 were not serious, and 287 had this criterion unknown. Where this data was available, reports had the seriousness criteria as disabling/incapacitating (998, 22.8%), followed by reports of reactions causing/prolonging hospitalization (579, 13.2%), or were reactions that were life-threatening (105, 2.4%), with 25 (0.6%) resulting in death. More than half of the reports (2959, 67.6%) had the seriousness criteria unknown. Of note is the fact that more than one seriousness criterion was possible per report. 

### 2.3. Adverse Drug Reactions’ Characteristics

Where available, both the time to onset of reaction and duration of reaction were mostly over 30 days, although a substantial proportion had a quicker onset (1–7 days) (Table 3). In most cases, with this information available, the drug was withdrawn, but the ADR did not resolve. Similarly, when rechallenge was performed, it showed recurrence of reaction in only a few cases (Table A1). Most ADRs were not recovered/resolved at the time of reporting, and five cases with peripheral neuropathy ended in patient death (with one death ruled not related to reaction) (Table 4).

### 2.4. Potential Risk Factors

With more than one concomitant medication possible per report, a total of 291 (6.3%) reports had nonsteroidal anti-inflammatory drugs (NSAIDs) listed as concomitant medication, with ibuprofen being the most frequently reported NSAID (131, 3.0% reports). Other drugs of interest (i.e., with possible interaction potential), such as theophylline or phenytoin, were reported as concomitant in 26 (0.6%) reports (theophylline, 22 and phenytoin, 4). Other drugs of interest (i.e., with possible interaction potential) were reported as concomitant in 63 (1.4%) reports.

Comorbidities were considered based on the indication field; a total of 51 (1.2%) reports had indications linked with diabetes of different etiologies (with more than one indication possible per report). Overall, a total of 112 (2.6%) reports had comorbidities with potential associated risk.

## 3. Discussion

Data retrieved from the clinical development of a new drug is oftentimes insufficient to assess its potential toxicity when used in real-world conditions. In turn, the post-marketing period becomes essential for consolidating drug safety profiles and detecting previously unknown ADRs [25,26]. Of great scientific value, spontaneous reporting systems (SRSs) can provide information about new drug-related adverse effects and even generate early signals. Analyzing such databases plays a primary role among pharmacovigilance (PV) methods [25]. To the extent of our knowledge, this is the first study aiming to evaluate FQ-induced peripheral nervous system disorders in VigiBase, the global database of spontaneous reports. A similar study was performed in the FDA Adverse Event Reporting System (FAERS) by Ali et al., evaluating potential quinolone-induced peripheral neuropathies reported up to 2012 [1]. The present evaluation of FQ-induced peripheral nervous disability in VigiBase tends to re-emphasize the link between FQs and PN, including post-referral PV data. Similar to all SRS databases, VigiBase encompasses advantages like continuous data collection and broad population coverage [27], thus representing one of the most valuable sources of PV data.

Our research included selected FQ-associated peripheral nervous system disorders reports up until July 2019, on which we performed descriptive analysis. Among these, a tendency of ADR reports to predominate in the female sex (54.0%) and adults aged between 18 and 64 years (49.8%) was observed. Similar sex distribution among reports was found by a literature search of FQ-induced neurological and psychiatric ADR case reports/series (i.e., 50.6% vs. 40.0%), with no statistically significant differences [28], as well by a study conducted in the spontaneous reporting Italian database (i.e., Rete Nazionale di Farmacovigilanza—RNF) including reports of musculoskeletal, neurological, or psychiatric ARDs (51% vs. 41%) [6]. In terms of age, in our dataset, the adults (i.e., 18–65 y.o.) were more frequently subject to FQAD [6,28], as opposed to the elderly. A similar gender and age distribution was observed by Ali et al [1]. The higher percentage of female reports experiencing FQ-induced disability could be attributed to gender differences in the pharmacokinetic and pharmacodynamic behavior of any drug [6], as well as women generally being more predisposed to neuropathic pain as opposed to men [29]. Moreover, the risk for women developing an ADR is 1.5- to 1.7-fold higher compared to men [30,31]. In turn, these results can be subject to bias, considering documented confounding factors, such as women generally reporting more [32], FQs being indicated in genitourinary infections, which are more common in females than in males [33], as well as the adult age group generally being predominant in our dataset, regardless of ADR. Of note is the fact that generally, FQs are contraindicated in the pediatric population.

Most of the reports originated from the US (82.7%), in line with a review noting that the US scored among the highest percentage of patient reports in 2014 (64%) [34]. Reporting frequency fluctuated, with a tendency to increase. The peak in reporting rates in 2016 could be a response to the safety issues brought to the public attention by the competent authority reviews (i.e., the Weber effect) [8].

FQ-related neurotoxicity leading to peripheral or sensory neuropathies has been documented early on [10], with further studies adding to the link between the two [1,35,36].

The CNS represents a target for quinolones [3,37,38], even more so for the fluorinated agents [39]. Early studies noted that quinolones have a structural resemblance to amfonelic acid, a CNS stimulant [40], thus implying a potential similar activity of the class. To date, several mechanisms of action at the core of the FQ-induced nervous system disorders have been proposed. More often than not, the inhibition of gamma-aminobutyric acid (GABA) receptor binding is invoked (GABA acting as an inhibitory transmitter in the CNS, inhibition of the same would have the opposite effect) [5,40,41,42,43,44]. Other studies suggest mechanisms involving interleukin-2 stimulation [42] or a dHLH protein DEC1 [3] being at the base of neurotoxicity caused by quinolones. However, it has been stated that there is no evidence for the association of the inhibition of cerebral GABA receptors (at the base of FQ-induced convulsions) being relevant to the pathogenesis of peripheral sensory disturbances. The pathological changes in drug-induced peripheral neuropathy consist of axonal degeneration with a secondary breakdown of the myelin sheath, or more rarely, primary segmental demyelination [45].

With regards to individually attributable toxicity, a consensus seems to be reached, noting that the disability-inducing potential is FQ agent-dependent [5,46]. Our results in VigiBase reveal levofloxacin to be mostly associated with ADR reports of interest, closely followed by ciprofloxacin and then moxifloxacin, in line with Ali et al.’s similar study in FAERS (levo- and ciprofloxacin being the topmost reported FQ, followed by moxifloxacin [1]), or the more recent Scavone et al. study (levofloxacin, 46.6% and ciprofloxacin, 40.5% [6]). Of note is the fact that we did not calibrate these results according to country- or region-dependent utilization/prescription patterns, as such data was not available, and they remain, therefore, subject to bias and should be interpreted as such.

Data regarding variables of ADRs, such as time to onset, duration, dechallenge/rechallenge action, and outcome, were scarce, and therefore no clear hypothesis can be drawn based on these results. Where available, both the time to onset and duration of reaction were, in most cases, between 1–7 days and ≥30 days. Despite data restraint, these results are in line with findings of other studies noting the highest incidence of peripheral sensory disturbances during the first days/weeks of treatment [1,41,45,47], but also after several months [28,47].

In our dataset, in most cases, the drug was withdrawn, but the ADR generally did not resolve, despite literature claims generally attesting remission of symptoms after treatment cessation [14,41,47]; positive dechallenge was also observed in a literature review of FQ-induced neurological and psychiatric ADR case reports/series, with the recovery of symptoms after one day [28]. However, in our study, the outcome for the evaluated reactions was not recovered/not resolved at the time of reporting, with percentages varying from 15 to 46% for different ADRs, adding to the disabling and potentially permanent aspect of FQ-induced peripheral nervous system disorders [10]. The vast majority of reports (81%) were serious, and for most reactions, the seriousness criteria were disabling/incapacitating (22.8%), followed by reports of reactions causing/prolonging hospitalization (13.2%).

Many of the FQ-associated ADRs occur more frequently in patients with pre-existing risk factors, or in certain subpopulations, and therefore could be prevented by improving patient screening and education [48]. To this extent, quinolones being commonly prescribed result in frequent combination with other drugs [49]. Potential drug interactions of quinolones with methylxanthine derivatives [2,4,13,43,50,51,52] and NSAIDs have been reported, resulting in considerable nervous system toxicity [13,43,50,51,52], as well as imipenem, foscarnet (phosphonoformic acid, Foscavir ^®^), cycloserine, fenbufen, and diphenylhydramine, which are known to increase the amplitude and frequency of the toxic CNS effects of FQs [52] if used simultaneously. Other studies also suggest a potential for drug-drug interaction with antacids [42,53], sucralfate, iron [53], nicotinamide, opiates (GABA-active substances) [54]. With more than one concomitant medication possible per report, a total of 291 (6.3%) reports had NSAIDs listed as concomitant medication, with ibuprofen being the most frequently reported NSAID (3.0%). Overall, approximately 10% of the reports had a concomitant potential interacting drug. Interpretation of this should be made with care since, in our particular dataset, concomitant drugs may not necessarily have been used at the same time as the suspected drug [27].

Other possible predisposing factors, such as impaired renal function (may lead to the increased serum concentration of the drug [44,45]), diabetes mellitus (may be associated with a lower threshold for drug-induced PN), or lymphatic malignancy (both due to neurotoxic antineoplastic therapy and the increased risk of infections leading to high antibiotic use) have been identified [45]. We, too, evaluated our dataset for such pre-existing comorbidities based on the indication field. Approximately 3% of the indications were represented by potentially conflicting disorders, half of which were represented by diabetes of different etiologies. This evaluation serves merely as a rough guide, since we could only base an idea on the indication field without taking into account a review (of any kind) of past history. Similarly, based on concurrent exposure to antidiabetic medication, Ali et al. found a low number of PN reports (N = 11) of patients with diabetes mellitus [1].

Nevertheless, at present, the benefit-risk balance stays positive for FQs; therefore, based on our results, as well as other safety studies, one should consider some practical aspects. Patient screening and education could be a useful tool in risk management, as well as in situations of particular nervous system concern; alternatives should be considered (e.g., cefuroxime axetil, macrolides, and co-amoxiclav [15].

### Limitations and Strengths

As with all SRSs, information regarding the true incidence of ADRs cannot be attained since such records are subject to under-reporting. However, we can assume under-reporting to be more or less of the same magnitude for the reference drugs [25]. Moreover, many studies based their assessment of drug safety on spontaneous reporting data and arguably offered information of great value. In addition, report quality in terms of inconsistent or missing information should always be considered; potential confounders, especially comorbidities or past patient history, are known limitations. Consequently, careful consideration should be granted to these aspects. In addition, access to information on the global use of a medicine can be limited. VigiBase reports cannot be treated as a random sample from a population of patients, as with clinical trials or observational studies. In a clinical trial, both the number of treated patients as well as the number of patients with a certain reaction is known [27].

Nevertheless, the results of our study not only add but strengthen the existing data, thus adding value to the continuous process of drug safety research and management.

## 4. Materials and Methods

### 4.1. Data Source

VigiBase, a database maintained by the Uppsala Monitoring Centre (UMC), with more than 19 million ICSRs submitted by national PV centers up to May 2019, was used as the data source. ICSRs encompass information on patient characteristics, ADRs, suspected drugs, seriousness, reporter type, year, and region. The reports originate from multiple sources, different countries, and types of reporters (e.g., healthcare professionals, consumers). As a result of the source diversity, a variation exists in terms of the amount of information in each report. Moreover, the probability that the suspected adverse effect is drug-related is not the same in all cases.

Nervous system disorders ADR reports (i.e., Medical Dictionary for Regulatory Activities [MedDRA] Preferred terms [PTs] grouped under the System Organ Class [SOC] Nervous system disorders associated with an FQ (ATC code: J01M) until 1 July 2019, were extracted from the UMC global database. In VigiBase, the reported reactions are coded according to the latest versions of the hierarchical structures of MedDRA (i.e., version 22.0 at the time of the search).

### 4.2. Data Selection and Analysis of Case Reports

Reports with an FQ (i.e., “WHO Drug preferred base name”) considered suspect or interacting in causing a nervous system disorder were included in the analysis. We specifically looked into ADRs pertaining to PN SMQ. SMQs are validated and pre-determined sets of MedDRA terms grouped together after extensive review and evaluation, aiming to facilitate retrieval of MedDRA-coded data as a first step in investigating drug safety issues in PV and clinical development [24]. Descriptive analysis was performed for the positive associations FQ-ADR (associated with at least 3 FQs, resulting from disproportionality analysis), for which we looked into general characteristics, as well as time to onset and duration of reaction, dechallenge/rechallenge actions and outcomes, seriousness and serious criteria, the outcome of the reaction, potentially associated risk factors, such as concomitant medication and disease.

### 4.3. Disproportionality Data Analysis

Disproportionality data analysis was performed overall for all nervous system FQ-related ADRs. Proportional reporting ratio (PRR) and information component (IC) (specifically developed and validated by UMC as a flexible, automated indicator value for disproportionate reporting) with their 95% confidence intervals (CIs) were used as provided by the UMC [27]. A PRR025 (lower end of the 95% CI for PRR) values >1 linked with ≥5 reports were considered as positive associations between the FQ and the ADR [55]. An IC025 value (lower end of the CI for IC) ≥0 was considered a positive FQ-ADR association. Extensive details on the IC and IC025 calculations were previously described [56].

## 5. Conclusions

Our results in VigiBase, the global database of ADR reports, strengthen the literature data related to FQ-induced PN. A tendency of female gender and adult population to prevail was observed, with the highest incidence of peripheral sensory disturbances occurring during the first days of treatment, or after several months. For up to 31% and 46% of cases of polyneuropathy and peripheral sensorimotor neuropathy, respectively, the outcome was not recovered/resolved, and in 23% of cases, the reaction was considered disabling/incapacitating.

## Figures and Tables

**Table 1:** Fluoroquinolone—Adverse drug reaction positive association *.

DrugText	ADR	No	IC	IC025	PRR	PRR025
Ciprofloxacin	Neuropathy peripheral	1946	2.55	2.48	6.00	5.74
Ciprofloxacin	Neuralgia	273	1.81	1.63	3.57	3.16
Ciprofloxacin	Peripheral sensorimotor neuropathy	13	2.11	1.22	5.06	2.92
Ciprofloxacin	Polyneuropathy	93	1.43	1.12	2.75	2.24
Ciprofloxacin	Sensory loss	44	0.77	0.31	1.72	1.28
Levofloxacin	Neuropathy peripheral	2302	3.01	2.95	8.31	7.97
Levofloxacin	Peripheral sensorimotor neuropathy	19	2.82	2.10	8.70	5.51
Levofloxacin	Polyneuropathy	85	1.52	1.19	2.92	2.36
Levofloxacin	Neuralgia	157	1.23	1.00	2.37	2.03
Levofloxacin	Sensory loss	35	0.65	0.14	1.59	1.14
Moxifloxacin	Neuropathy peripheral	1243	3.36	3.28	10.46	9.90
Moxifloxacin	Peripheral sensorimotor neuropathy	8	2.55	1.38	8.50	4.23
Moxifloxacin	Polyneuropathy	41	1.68	1.21	3.32	2.44
Moxifloxacin	Neuralgia	52	0.87	0.45	1.85	1.41
Moxifloxacin	Sensory loss	20	1.06	0.36	2.15	1.38

ADR: adverse drug reaction; IC: information component; No: number of reports; PRR: proportional reporting ratio; * PRR025 (lower end of the 95% CI for PRR) value >1 associated with ≥5 cases were considered a positive association between the FQ and the ADR; IC025 (lower end of the CI for IC) ≥0 was considered a positive FQ-ADR association.

**Table 2:** General characteristics of reports.

	Number of Reports N = 4374, %		N/n %
***Sex***		***Reporting years***	***N = 4374***
Female	2364, 54.1	1987–1994	64, 1.5
Male	1483, 33.9	1995–1999	51, 1.2
Unknown	527, 12.1	2000–2004	139, 3.2
***Age, years***		2005–2009	310, 7.1
<18	31, 0.7	2010–2014	635, 14.5
18–64	2175, 49.7	2015–2019	3175, 72.6
≥65	458, 10.5		
Unknown	1710, 39.1	ADR *	***n = 6331***
***UN Continent***		Neuropathy peripheral	5492, 86.7
America	3617, 82.7	Neuralgia	481, 7.6
Europe	612, 14.0	Polyneuropathy	220, 3.5
Asia	100, 2.3	Sensory loss	99, 1.6
Africa	20, 0.5	Peripheral sensorimotor neuropathy	39, 0.6
Oceania	25, 0.6		
Reporter *	***n = 9634***	FQ suspect *	***n = 6331***
HCP	4010, 41.6	Levofloxacin	2597, 41.0
Consumer	4237, 44.0	Ciprofloxacin	2373, 37.5
Other	711, 7.4	Moxifloxacin	1361, 21.5
Unknown	676, 7.0		

ADR: adverse drug reaction; FQ: fluoroquinolone; N: number of reports; n: number of adverse drug reactions; * more than one reporter/fluoroquinolone/adverse drug reaction possible per report.

**Table 3:** Time to onset and duration of reaction.

	Time to Onset (Days), % *					Duration of Reaction (Days), % *				
ADR	1–7	8–14	15–29	≥30	NA	1–7	8–14	15–29	≥30	NA
Neuropathy peripheral (N = 5492)	69, 1.3	11, 0.2	30, 0.5	95, 1.7	5287, 96.3	12, 0.2	2	4, 0.1	16, 0.3	5458, 99.4
Neuralgia (N = 481)	19, 4.0	3, 0.6	4, 0.8	13, 2.7	442, 91.9	7, 1.5	0	0	8, 1.7	466, 96.9
Polyneuropathy (N = 220)	14, 6.4	5, 2.3	3, 1.4	13, 5.9	185, 91.9	0	2, 0.9	1, 0.5	3, 1.4	214, 97.3
Sensory loss (N = 99)	3, 3.03	0	2, 2.0	2, 2.0	92, 92.9	2, 2.0	0	0	0	97, 98.0
Peripheral sensorimotor neuropathy (N = 39)	0	0	1, 2.6	1, 2.6	37, 94.9	0	0	0	0	39, 100.0

ADR: adverse drug reaction; N: number of adverse drug reactions; NA: not available. * Only percentages above 0.1 were included in the table.

**Table 4:** Outcome of adverse drug reactions.

	Outcome, %					
ADR	Not Recovered/Not Resolved	Recovered/Resolved	Recovered/Resolved with Sequelae	Recovering/Resolving	Fatal	NA
Neuropathy peripheral * (N = 5492)	1390, 25.3	71, 1.3	33, 0.6	104, 1.9	5, 0.1	3889, 70.8
Neuralgia (N = 481)	93, 19.3	19, 4.0	14, 2.9	19, 4.0	-	336, 69.9
Polyneuropathy (N = 220)	69, 31.4	19, 8.6	8, 3.6	14, 6.4	-	110, 50.0
Sensory loss (N = 99)	15, 15.2	5, 5.1	-	9, 9.1	-	70, 70.7
Peripheral sensorimotor neuropathy (N = 39)	18, 46.2	2, 5.1	-	-	-	19, 48.7

ADR: adverse drug reaction; N: number of adverse drug reactions; NA: not available. * in 1 case, the outcome was fatal, but not related to reaction.

## Data Availability

Data is contained within the article.

## Appendix A

**Table A1:** Dechallenge/rechallenge action and outcome.

ADR	Drug Withdrawn	Dose not Changed	Dose Reduced	NA	ADR	Rechallenge	No Rechallenge	NA
Neuropathy peripheral (N = 5492)	***564*** *(10.3)*	*81 (1.5)*	*1 (0.0)*	*4846 (88.2)*	Neuropathy peripheral (N = 5492)	***1194*** *(21.7)*	*26 (0.5)*	*4272 (77.8)*
*No effect observed*	*328 (58.2)*				*No recurrence*	*110 (9.2)*		
*Reaction abated*	*108 (19.1)*				*Reaction recurred*	*4 (0.3)*		
*NA*	*128 (22.7)*				*NA*	*1080 (90.5)*		
Neuralgia (N = 481)	***148*** *(30.8)*	*7 (1.5)*	*2 (0.4)*	*324 (67.4)*	Neuralgia (N = 481)	***43*** *(8.9)*	*5 (1.0)*	*433 (90.0)*
*No effect observed*	*100 (67.6)*				*No recurrence*	*1 (2.0)*		
*Reaction abated*	*28 (18.9)*				*Reaction recurred*	*2 (3.9)*		
*NA*	*20 (13.5)*				*NA*	*48 (94.1)*		
Polyneuropathy (N = 220)	***70*** *(31.8)*	*14 (6.4)*	*2 (0.9)*	*134 (60.9)*	Polyneuropathy (N = 220)	***51*** *(23.2)*	*-*	*169 (76.8)*
*No effect observed*	*25 (35.7)*				*No recurrence*	*13 (30.2)*		
*Reaction abated*	*17 (24.3)*				*Reaction recurred*	*2 (4.7)*		
*NA*	*28 (40.0)*				*NA*	*28 (65.1)*		
Sensory loss (N = 99)	***28*** *(28.3)*	*3 (3.0)*	*1 (1.0)*	*67 (67.7)*	Sensory loss (N = 99)	***12*** *(12.1)*	*1 (1.0)*	*86 (86.9)*
*No effect observed*	*12 (42.9)*				*No recurrence*	*1 (8.3)*		
*Reaction abated*	*11 (39.3)*				*Reaction recurred*	*-*		
*NA*	*5 (17.9)*				*NA*	*11 91.7()*		
Peripheral sensorimotor neuropathy (N = 39)	***3*** *(7.7)*	*-*	*-*	*36 (92.3)*	Peripheral sensorimotor neuropathy (N = 39)	***12*** *(30.8)*	*-*	*27 (69.2)*
*No effect observed*	*1 (33.3)*				*No recurrence*	*2 (16.7)*		
*Reaction abated*	*-*				*Reaction recurred*	*2 (16.7)*		
*NA*	*2 (66.6)*				*NA*	*8 (66.7)*		
